# Relationship among Insulin Therapy, Insulin Resistance, and Severe Coronary Artery Disease in Type 2 Diabetes Mellitus

**DOI:** 10.1155/2022/2450024

**Published:** 2022-09-26

**Authors:** Jing Song, Xinyi Xia, Ye Lu, Jing Wan, Haibing Chen, Jun Yin

**Affiliations:** ^1^Department of Endocrinology and Metabolism, Shanghai Eighth People's Hospital, Shanghai, China; ^2^Department of Endocrinology and Metabolism, Shanghai Jiao Tong University School of Medicine Affiliated Sixth People's Hospital, Shanghai Clinical Center for Metabolic Diseases, Shanghai Key Laboratory of Diabetes Mellitus, Shanghai, China; ^3^Department of Endocrinology and Metabolism, Shanghai 10th People's Hospital, Tongji University, Shanghai, China

## Abstract

**Objectives:**

The effect of insulin therapy on coronary artery disease (CAD) remains controversial. This study aimed to analyze the association between insulin resistance and the morbidity of severe CAD in type 2 diabetes mellitus (T2DM).

**Methods:**

A total of 2044 T2DM patients aged ≥40 years were included in this cross-sectional observational study. Clinical information and laboratory results were collected from the medical records. Those who underwent percutaneous coronary intervention (PCI) were classified as severe CAD, while those who did not have a history of and were not suffering from CAD were classified as patients without CAD.

**Results:**

T2DM patients with severe CAD and without CAD had no significant differences in glycosylated hemoglobin A1c (8.55% ± 2.10% vs. 8.39% ± 1.77%, *P*=0.234). The proportion of insulin treatment was also similar between the two groups (56.85% *vs*. 53.65%, odds ratio = 1.138, *P*=0.310). In the patients without insulin treatment, the levels of fasting C peptide (FCP) correlated with severe CAD prevalence. FCP was categorized into 3 tertiles (<1.5 ng/mL, 1.5 ng/mL- 3 ng/mL, and ≥3 ng/mL), and the prevalence rates of severe CAD were 7.88%, 14.31%, and 18.28%, respectively (*P* < 0.05). In the patients with insulin treatment, the body mass index (BMI) was the significant risk factor of severe CAD. The prevalence of severe CAD according to BMI tertiles (<24 kg/m^2^, 24 kg/m^2^–28 kg/m^2^, and ≥28 kg/m^2^) was 11.22%, 14.61%, and 24.62%, respectively (*P* < 0.01).

**Conclusions:**

Our results showed that insulin resistance, rather than insulin therapy, increases the risk of severe CAD in T2DM patients with inadequate glycemic control. Non-insulin treated patients with high FCP and insulin-treated patients with high BMI are at higher risk of severe CAD.

## 1. Introduction

Diabetes mellitus (DM) is a worldwide epidemic disease that presents an increasing trend. Cardiovascular disease is a major macrovascular complication of type 2 diabetes mellitus (T2DM). Multiple antihyperglycemic medications are used for glycemic control to prevent diabetic complications. Of all antihyperglycemic medications, insulin has the greatest capacity to lower blood glucose and is widely used in routine clinical practice for T2DM.

However, the effect of insulin therapy on cardiovascular outcomes in patients with T2DM remains controversial. Some studies suggested that insulin therapy in T2DM increased, rather than decreased, the risk of cardiovascular events [1, 2]. Endogenous hyperinsulinemia and exogenous insulin are a common theme of increased cardiovascular risk [3], but their respective effects on cardiovascular disease have not been compared in one study. In addition, the plasma insulin level is easily interfered by exogenous insulin injection. Thus, we aimed to investigate relationship among insulin therapy, plasma insulin/C-peptide levels, and the morbidity of severe coronary artery disease (CAD) in T2DM.

## 2. Materials and Methods

### 2.1. Study Population

This was a cross-sectional study of 2044 patients with poorly controlled T2DM who were hospitalized in the Department of Endocrinology and Metabolism of Shanghai Jiao Tong University School of Medicine Affiliated Sixth People's Hospital, between January 2011 and November 2012. We recorded the patients' detailed medical history, including the duration of diabetes, history of hypertension, and administered drugs. T2DM was defined according to 2012 American Diabetes Association criteria [4].

The exclusion criteria were type 1 diabetes mellitus, acute complications of diabetes, and liver and renal dysfunction. Since this study was a retrospective analysis, we were unable to accurately determine whether the patients with stable angina, who did not undergo coronary angiography, had CAD or not, and therefore, these patients were excluded. The patients who diagnosed as CAD by coronary angiogram but did not undergo PCI were also excluded as we could not determine whether their diseases progressed to need PCI at the time of hospitalization for diabetes.

### 2.2. Ethical Approval

This study was approved by the Human Research and Ethics Committee of Human Research and Ethics Committee of Shanghai Jiao Tong University School of Medicine Affiliated Sixth People's Hospital and was conducted according to the tenets of the 1964 Declaration of Helsinki and its later amendments. Written informed consent was obtained from all participants.

### 2.3. Diagnostic Criteria

Diabetic patients were classified as insulin-treated diabetes mellitus (ITDM) if they were taking insulin alone or with oral antihyperglycemic agents for ≥3 months before admission or as noninsulin-treated diabetes mellitus (NITDM) if they were treated with oral antihyperglycemic agents and/or lifestyle modifications. Exogenous insulin administrated was commercial human insulin. Glycemic control was determined by HbA1c levels. Hypertension was defined as systolic blood pressure ≥140 mmHg, diastolic blood pressure ≥90 mmHg, or use of antihypertensive medications. Those who underwent PCI were classified as patients with severe CAD, whereas those who did not have a history of and were not suffering from CAD were classified as patients without CAD.

### 2.4. Anthropometric and Biochemical Measurements

Height, weight, and blood pressure were recorded. Body mass index (BMI) was calculated as weight (kg) divided by height squared (m^2^). Obesity was defined as BMI ≥30 kg/m^2^ and overweight was defined as BMI ≥25 kg/m^2^ and <30 kg/m^2^. Blood samples were obtained after a 12-hour fast without medication. The plasma levels of lipids were measured by enzymatic methods using an automatic biochemical analyzer (7600–020; Hitachi Inc., Tokyo, Japan). HbA1c was measured by high-pressure liquid chromatography using the Variant™ II machine (Bio-Rad, Hercules, CA, USA). Serum insulin and C-peptide levels were measured using a Cobas analyzer (Roche, Switzerland).

### 2.5. Statistical Analysis

Categorical variables were presented as numbers and relative frequencies (percentages) and continuous variables as means and standard deviations or medians with interquartile ranges (Q1-Q3). Standard *t* test and chi-square test were used for continuous and categorical variables, respectively. A skewed distribution (assessed by the Kolmogorov–Smirnov test) was presented as median with 25th to 75th percentiles and compared using the Kruskal–Wallis rank-sum test. Binary logistic regression models were used to assess the correlates of CAD in the non-insulin-treated and insulin-treated groups, respectively. Statistical analyses were performed using SPSS version 23.0 (IBM Corp., Armonk, NY, USA). A two-tailed *p* value < 0.05 was considered statistically significant.

## 3. Results

### 3.1. Basic Characteristics of the Subjects

The clinical characteristics of the patients are presented in Table 1. T2DM patients with severe CAD were older and had higher proportion of hypertension, longer duration of diabetes, higher body mass index (BMI), higher fasting insulin (FINS), and fasting C peptide (FCP). There were no significant differences in sex, glycemic control indicated by fasting blood glucose, and HbA1c.

### 3.2. Comparison between Patients with Severe CAD and without CAD

In our study, the proportion of severe CAD did not differ between NITDM and ITDM (13.43% vs. 15.01%, *P*=0.310). We further investigated the risk factors of severe CAD in the NITDM and ITDM groups, respectively. In either NITDM or ITDM patients, age, proportion of hypertension, diabetes duration, FINS and FCP were higher in those with severe CAD. Only ITDM patients with severe CAD had a higher BMI level (Table 1).

### 3.3. Relationship of Severe CAD with BMI, FINS, and FCP

**Figure 1:**
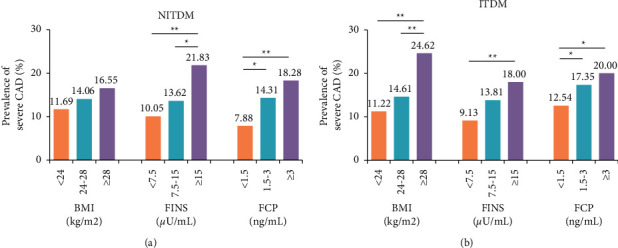
Prevalence of severe CAD in (a) NITDM and (b) ITDM patients according to BMI, FINS, and FCP. ^*∗*^*P* < 0.05 and ^*∗∗*^*P* < 0.01. BMI, body mass index; CAD, coronary artery disease; FCP, fasting C peptide; FINS, fasting insulin; ITDM, insulin-treated diabetes mellitus; NITDM, non-insulin-treated diabetes mellitus.

BMI, FINS, and FCP were categorized into 3 tertiles (Figure 1). The prevalence of severe CAD significantly increased across FINS and FCP tertiles in both groups, while only in ITDM, a higher prevalence of severe CAD was observed in overweight and obese patients (*P* < 0.01).

**Figure 2:**
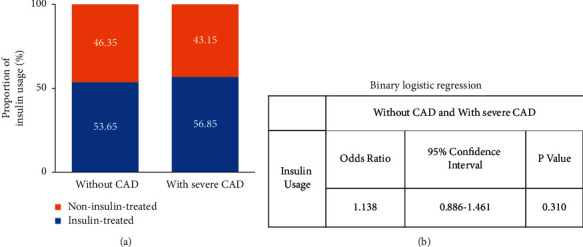
Analysis of insulin usage in patients with T2DM with severe CAD and without CAD. (a) Proportion. (b) Binary logistic regression. CAD, coronary artery disease.

The proportion of insulin therapy was similar in patients with severe CAD and without CAD. Binary logistic regression was performed to assess the association between insulin therapy and severe CAD in all patients, showing that insulin treatment was not related to severe CAD (Figure 2). Then, the relation of relevant risk factors to severe CAD in the NITDM and ITDM groups was investigated, respectively. The variables, which were statistically different between patients with severe CAD and without CAD in Table 1, were analyzed in the logistic regression model. The correlation among age, hypertension, and severe CAD was statistically significant in both subgroups. The duration of diabetes did not correlate with severe CAD in both groups. FCP, instead of BMI, were positively associated with increased risk of severe CAD in the NITDM group (*P*=0.038). On the contrary, BMI was associated with increased risk of severe CAD in the ITDM group (*P* < 0.001), rather than FINS and FCP (Table 2).

## 4. Discussion

This study analyzed the association among insulin therapy, FINS/FCP levels, and the risk of severe CAD in patients with T2DM. The results showed that exogenous insulin therapy was not the risk factor of severe CAD in patients with T2DM. Previous studies have drawn different conclusions on the effects of insulin on cardiovascular outcomes. Insulin treatment has been shown to benefit cardiovascular outcomes in some studies but not in others [1, 2, 5]. Recently, a meta-analysis proposed that insulin therapy increased the cardiovascular risk and mortality among T2DM patients in several reported clinical trials, such as ADVANCE, ACCORD, and VADT [6]. However, not all of the subjects in the intensive arm of the 3 studies were treated with insulin (40%, 77%, and 89%, respectively), which was different from our insulin-treated group. After intensive therapy, the average HbA1c levels were 6.4% in ACCORD and ADVANCE and 6.9% in VADT [7–9]. None of the intensive-therapy groups in these studies had reduced cardiovascular events, even the ACCORD study was terminated at 3.5 years because of increased mortality [9]. The rates of hypoglycemia and weight gain were greater in the intensive-therapy group in the three trials. So, it was considered that strict glucose control may increase the side effects of insulin, which covered the real effect of insulin treatment on CAD [10]. An important observation from early study revealed that mortality associated with insulin treatment followed a U-shaped distribution [11]. In our study, although there was no significant difference in the average HbA1c level in patients with severe CAD and without CAD (8.55% ± 2.10% vs. 8.39% ± 1.77%, *P*=0.18), which excluded the influence of blood glucose on cardiovascular events, we found no difference in the proportion of severe CAD between the NITDM and ITDM groups. Combined with the result of binary logistic regression, this study indicates that insulin therapy has a little adverse effect on severe CAD in T2DM with inadequate glycemic control, which suggests few severe hypoglycemic episodes.

In the subgroup analysis, FINS and FCP levels in the NITDM group and BMI in the ITDM group were associated with severe CAD. Previous studies also showed that elevated FINS levels were significantly associated with the risk of CAD, but these observations were mainly from a nondiabetic population [12]. Our results showed that the prevalence of severe CAD increased gradually when FINS exceeded 7.5 *µ*U/mL in NITDM patients, especially when exceeding 15 *µ*U/mL, the severe CAD prevalence increased 2 times. Same trend can be found on FCP in NITDM patients too. Similar correlation was also observed in ITDM patients whose insulin function was supposed to be poor. However, after logistic regression, BMI was proved to be significantly associated with severe CAD. Overweight and obesity increased the severe CAD risk, and the risk of severe CAD in obese patients was more than double. In this study, either hyperinsulinemia or BMI was associated with severe CAD, which confirmed the relationship between insulin resistance and severe CAD.

Insulin resistance is an important pathogenesis of T2DM and is closely related to cardiovascular adverse events. Hence, the “common soil” theory, which means insulin resistance is the common basis of T2DM and cardiovascular events, has been put forward [13]. However, it is difficult to choose simple clinical parameters to reflect the extent of insulin resistance, especially in patients receiving insulin treatment. For the patients with poor glucose control, homeostasis model assessment for insulin resistance (HOMA-IR), quantitative insulin sensitivity check index (QUICKI), and such may not be applicable under the interference from high blood glucose, impaired islet function and antihyperglycemic drugs. In this study, we found that BMI was positively correlated with severe CAD risk rather than FINS in ITDM patients. In these patients, the insulin level was interfered by exogenous insulin and the C-peptide level was limited by islet dysfunction, which cannot reflect the extent of insulin resistance. Thus, only BMI could predict the risk of severe CAD as a parameter of insulin resistance in ITDM patients.

There are some limitations in the present study. First, since the included patients were hospitalized, their glycemic control is not adequate at admission. Second, as the data were obtained from the hospital information system, there were varying degrees of missing information, such as smoking history, the time when the PCI was performed, detailed information of insulin usage including duration, dose and type of insulin, and medications affecting CAD. Thus, the information was not included in the analysis. Third, this is a retrospective analysis; hence, the prospective studies covered both outpatients and inpatients are needed to confirm the relationship between insulin therapy and risk of CAD.

In conclusion, our results showed that insulin therapy did not increase the risk of severe CAD in T2DM patients with an inadequate glycemic control. NITDM patients with high FCP and ITDM patients with overweight or obesity were at a higher risk of severe CAD. These findings have important implications for CAD prevention in T2DM.

## Figures and Tables

**Table 1:** Clinical characteristics of patients with T2DM with severe CAD and without CAD.

Variable	*All patients*			*NITDM*			*ITDM*		
	Without CAD (*n* = 1752)	With severe CAD (*n* = 292)	*P* value	Without CAD (*n* = 812)	With severe CAD (*n* = 126)	*P* value	Without CAD (*n* = 940)	With severe CAD (*n* = 166)	*P* value
Age (years)	61.05 ± 10.12	66.36 ± 9.37	<0.001	61.11 ± 10.13	65.75 ± 9.27	<0.001	60.99 ± 10.12	66.82 ± 9.45	<0.001
Men	982 (56.05)	158 (54.11)	0.537	454 (55.91)	68 (53.97)	0.683	528 (56.17)	90 (54.22)	0.640
Hypertension	950 (54.24)	214 (73.36)	<0.001	450 (55.42)	91 (72.22)	<0.001	500 (53.19)	123 (74.10)	<0.001
Duration of diabetes	10.34 ± 6.75	12.43 ± 6.97	<0.001	9.02 ± 6.23	10.21 ± 5.86	0.044	11.47 ± 6.97	14.10 ± 7.29	<0.001
Body mass index (kg/m^2^)	24.85 ± 3.47	25.78 ± 3.31	<0.001	24.77 ± 3.40	25.40 ± 3.24	0.057	24.91 ± 3.53	26.07 ± 3.34	<0.001
Total cholesterol (mmol/L)	4.81 ± 1.10	4.48 ± 1.23	<0.001	4.78 ± 1.07	4.35 ± 1.14	<0.001	4.84 ± 1.13	4.59 ± 1.28	0.015
Triglycerides (mmol/L)	1.75 ± 1.47	1.69 ± 1.45	0.512	1.71 ± 1.34	1.58 ± 0.93	0.289	1.79 ± 1.58	1.78 ± 1.75	0.951
HDL (mmol/L)	1.12 ± 0.31	1.09 ± 0.29	0.056	1.11 ± 0.29	1.09 ± 0.29	0.343	1.13 ± 0.32	1.08 ± 0.29	0.090
LDL (mmol/L)	2.85 ± 0.87	2.61 ± 0.93	<0.001	2.89 ± 0.85	2.53 ± 0.87	<0.001	2.83 ± 0.88	2.68 ± 0.97	0.062
FBG (mmol/L)	7.99 ± 2.73	7.70 ± 2.73	0.087	7.81 ± 2.54	7.36 ± 2.16	0.058	8.15 ± 2.88	7.95 ± 3.08	0.427
Hemoglobin A1c (%)	8.55 ± 2.10	8.39 ± 1.77	0.234	8.34 ± 2.06	8.07 ± 1.66	0.182	8.74 ± 2.11	8.64 ± 1.82	0.609
Fasting insulin (*μ*U/mL)	10.68[6.72–18.15]	13.73[8.74–24.43]	<0.001	8.64 [5.86–12.10]	10.57 [7.16–15.02]	<0.001	14.82 [8.45–27.64]	19.41 [11.76–39.13]	<0.001
Fasting C peptide (ng/mL)	1.85 ± 1.07	2.13 ± 1.25	<0.001	2.16 ± 0.99	2.57 ± 1.25	<0.001	1.58 ± 1.07	1.80 ± 1.15	0.009

Values are presented as mean ± standard deviation, *n* (%), or median (interquartile range). Standard *t* test, Kruskal–Wallis rank-sum test, and chi-square test were performed according to the type of variables, respectively. CAD, coronary artery disease; FBG, fasting blood glucose; HDL, high-density lipoprotein; ITDM, insulin-treated diabetes mellitus; LDL, low-density lipoprotein; NITDM, non-insulin-treated diabetes mellitus.

**Table 2:** Binary logistic regression coefficients for association between NITDM or ITDM with severe CAD and without CAD.

Variables^†^	*NITDM with severe CAD and without CAD*			Variables^†^	*ITDM with severe CAD and without CAD*		
	Odds ratios	95%CI	*P* value		Odds ratios	95%CI	*P* value
Hypertension	1.600	1.025–2.500	0.039	Hypertension	1.624	1.094–2.411	0.016
Fasting C peptide (ng/mL)	1.216	1.011–1.462	0.038	Body mass index (kg/m^2^)	1.104	1.051–1.159	<0.001
Age (years)	1.042	1.021–1.063	<0.001	Age (years)	1.060	1.040–1.079	<0.001
Fasting insulin (*μ*U/mL)	1.028	0.999–1.057	0.056				

^†^ Age, hypertension, body mass index, duration of diabetes, fasting insulin and fasting C peptide are analyzed in the multivariate model, and those in the equation in multiple model are shown in the table. CAD, coronary artery disease; CI, confidence interval; NITDM, non-insulin-treated diabetes mellitus, ITDM, insulin-treated diabetes mellitus.

## Data Availability

The data used to support the findings of this study are available on reasonable request to corresponding author.
